# Sickness absence and self-reported health a population-based study of 43,600 individuals in central Sweden

**DOI:** 10.1186/1471-2458-8-426

**Published:** 2008-12-30

**Authors:** Hans-G Eriksson, Anna-Sophia von Celsing, Rolf Wahlström, Lotta Janson, Viktoria Zander, Thorne Wallman

**Affiliations:** 1 R&D Centre/Centre for Clinical Research, Sörmland County Council, SE-631 88 Eskilstuna, Sweden; 2 Department of Public Health Sciences, Karolinska Institutet, Stockholm, Sweden; 3 Department of Public Health and Caring Sciences, Family Medicine and Clinical Epidemiology Section, Uppsala University, Uppsala, Sweden

## Abstract

**Background:**

Sickness absence is very high in Sweden. The reasons for this phenomenon are not well known. The aim of this study was to investigate the association between degree of self-reported sickness absence and health. The hypothesis was that individuals with long-term sickness absence would report more symptoms and lower self-rated health. Another hypothesis was that women are more likely to self-rate psychiatric diagnoses compared to men, who are more likely to self-rate musculoskeletal diagnoses.

**Methods:**

The data was obtained with a postal survey questionnaire answered by 43,589 individuals, a Swedish random population sample of men and women aged 18–84 years. The response rate was 65%. This study included 19,826 individuals aged 18–64 years old and still at work. They were divided into four groups, based on the number of reported days of sickness absence during the past year.

**Results:**

Approximately 40% of the individuals at work mentioned that they had been absent due to illness sometime during the past year. Of those who had been absent 90 days or more, two thirds were women. There was a significant difference between the groups in self-rated health (p < 0.05). Every fifth woman (19.4%) and every fourth man (25.9%) in the group with a sickness absence of more than 89 days rated their health as poor or very poor, but a large proportion, 43.5% of the women and 31.6% of the men, rated their health as good. Long-term illnesses and complaints differed between the groups. The correlations between the groups and illness were mostly significant (p < 0.01). Two thirds of the subjects had both psychiatric and musculoskeletal symptoms. There was a significant difference among them, as men more often had musculoskeletal diagnoses. One third had only psychiatric or musculoskeletal symptoms and in those groups there were no significant diagnosis differences between the sexes.

**Conclusion:**

Individuals with long-term sickness absence reported more symptoms and lower self-rated health than did those who had not been absent at all, and than those who had been ill 1–28 days. Men and women sick-listed 29 days or more generally reported more illness and complaints. No sex differences among psychiatric and musculoskeletal diagnoses were found, but when reported both psychiatric and musculoskeletal symptoms the musculoskeletal diagnoses were significant among men.

## Background

Sickness absence is very high in Sweden. In December 2006 the number of ongoing cases of sickness cash benefit per 1,000 persons insured was 46.9 for woman and 25.9 for men [1]. The reasons behind sickness absence are not well known. Statistics show that women have a higher degree of sickness absence than men. Long-term sickness absence increases by age. Musculoskeletal and psychiatric diagnoses dominate. We also know that long-term sickness absence is a risk marker for future disability pension [2].

There are only a few population-based studies on self-reported health related to sickness absence [3-8]. The aim of this study was to investigate the association between degree of self-reported sickness absence and health, with the hypothesis that individuals with long-term sickness absence would report more symptoms and lower self-rated health. Sick leave may provide an important risk marker for identifying groups at high risk of a disability pension, especially for psychiatric diagnoses [9].

Another hypothesis was that women are more likely to self-rate psychiatric diagnoses than are men, who are more likely to self-rate musculoskeletal diagnoses. Official statistics on sick-leave days from The Swedish Social Insurance Agency 2004, show that among women it is more common with psychiatric diagnoses and among men it is more common with musculoskeletal diagnoses[10].

## Methods

In order to improve the information needed for planning, distribution of resources and management, five county councils in central Sweden (Sörmland, Uppsala, Värmland, Västmanland and Örebro) have collaborated in the field of social medicine, using existing resources and the existing competence within assigned units in the different county councils.

During the fall of 2004, 43,589 individuals in these five counties answered the Liv & hälsa [11] inquiry about their personal health, sickness absences, living conditions, ways of living and their contacts with medical services. The EQ-5D instrument was used. This is the largest investigation of residents in central Sweden. The data was obtained using a postal survey questionnaire, with fixed list answers, sent to a random population sample of men and women aged 18–84 years. The sampling was random and stratified by gender, age group, county and municipality. The data collection was completed after two postal reminders. The overall response rate was 64%, with only small differences between the counties (± 2%). The area investigated covers 55 municipalities with a total of about 1 million inhabitants in the age group.

### Study design and measures

In this study a cross-sectional survey design was used. The analysis is based on a classification that divides people at work, aged 18–64, and based on the variable "length of sickness absence". This variable was divided into four classes already in the inquiry due to self-reported illness during the past year. In Sweden the old age pension level is 65 years of age. Group 1 included those who had not been absent at all (11,756 individuals (59%)), Group 2 comprised individuals who had reported sickness absence during 1–28 days (6,280 individuals (32%), Group 3 comprised individuals who had reported absence 29–89 days (945 individuals (5%)) and Group 4 included those with reported absence 90 days or more (845 individuals (4%)). In this study we define long-term sickness absence as more than 28 days (Groups 3 and 4).

In the International Classification of Diseases, version 10 (ICD10) [12], psychiatric diagnoses are termed F and musculoskeletal diagnoses are termed M. F diagnoses were defined as reported sickness absence because of burn-out syndrome, depression, stress or other psychiatric problems including sleeping disorders. M diagnoses were defined as reported sickness absence because of complaints from back, neck, joints or muscles.

The diagnosis group with the greatest increase in sickness absence in Sweden today is that of the psychiatric diagnoses.

Out of the study population, we identified those sick-listed 29 days or more during last twelve months (1790 individuals), and studied their answers of the question why they were on sickness absence. Only those with musculoskeletal or/and psychiatric diagnosis, and who had answered the questions about complaints and symptoms were included (864 individuals). Pain in neck and shoulders, or in back or hips, or in arms, legs, knees or feet, were considered to be musculoskeletal symptoms. Anxiety, tiredness and weakness, sleeping disorders and depression were considered to be psychiatric symptoms. A total of 800 people satisfied all demands.

The questions about self-rated health and EQ-5D are both validated and internationally acknowledged [13,14]. EQ-5D is an instrument to describe and value health [13]. It is a standardised instrument for use as a measure of health outcome. There seems to be an increasing demand for EQ-5D usage in population health surveys [14]. EQ-5D was initially developed simultaneously in Dutch, English, Finnish, Norwegian and Swedish.

The symptoms and complaints used in Tables three and four were selected by a number of medical advisers and experts in epidemiology and statistics in this geographic area. The respondents answered fixed alternatives on a list with symptoms and complaints during the last three months. The two alternatives in table three were "No" and "Yes". The four alternatives in table four were "No symptoms", "Occasionally", "Several times", "Most of the time". The proportions of the last two alternatives are summarized in the table.

### Study population

Out of the 43,589 individuals between 18 and 84 of age that answered the Liv & hälsa inquiry [11] in 2004, 20,140 were at work and between 18 and 64 of age. Out of that group 98.4% (19,826 individuals 10,275 women, 9,551 men) responded to questions regarding sickness absence during the past twelve months.

### Statistical methods

The major part of the article consists of descriptive data, calibrated to erase fall-offs using a method from Statistics Sweden, and weighted to be equivalent to the population in the area [15]. Tests of correlations (Spearman) and also some tests of independence (Pearson Chi-square and Fischer's exact test) have been carried out with SPSS (The Statistical Package for Social Sciences, version 14.0).

In Tables three and four the issue of statistical multiple test effect arises, but by choosing 0.01 as significance level that potential bias will, to some extent, be taken into account.

## Results

### Sickness absence, life and health

Approximately 40 percent of the individuals at work mentioned that they had been absent due to illness sometime during the last year. Out of that group, every tenth individual had been absent due to illness on a long-term basis, more than 28 days. Of those who had been absent 90 days or more, two thirds were women (Table 1).

**Table 1:** Description of the study population (n = 19826).

	Group 1	Group 2	Group 3	Group 4
N	11756 (59.3%)	6280 (31.7%)	945 (4.8%)	845 (4.3%)
Women (%)	47.6	56.0	63.0	68.2
Men (%)	52.4	44.0	37.0	31.8
Age, average	45.3	42.6	45.2	48.2
Age, median	47.0	42.0	46.0	50.0

Unweighted data

### Self-rated health

As hypothesised, there was a significant difference between these groups concerning self-rated health (p < 0.05, Spearman). The first question was: "How do you rate your general health?"

Every fifth woman (19.4%) and every fourth man (25.9%) in Group 4 rated their health as poor or very poor, but a large proportion, 43.5% of the women and 31.6% of the men, rated their health as good or very good. This is a difference compared to Group 1, where slightly more than one percent of both women and men rated their health as poor or very poor and as many as 86,8% of the women and 84,9% of the men rated their health as good or very good (Table 2).

**Table 2:** Distribution of self-rated health (percent of all in each group).

	Women				Men			
Options	Gr 1	Gr 2	Gr 3	Gr 4	Gr 1	Gr 2	Gr 3	Gr 4
Very good	29.6	15.4	10.2	4.2	27.0	18.7	12.6	3.0
Good	57.2	62.1	50.1	39.3	57.9	58.6	44.3	28.6
Neither good nor poor	11.8	19.9	29.6	37.2	13.8	19.5	31.8	42.6
Poor	1.3	2.3	9.7	18.2	1.3	3.1	10.1	23.9
Very Poor	0.0	0.2	0.4	1.2	0.1	0.1	1.2	2.0

Age-standardised and weight-calibrated.

### EQ-5D

**Figure 1:**
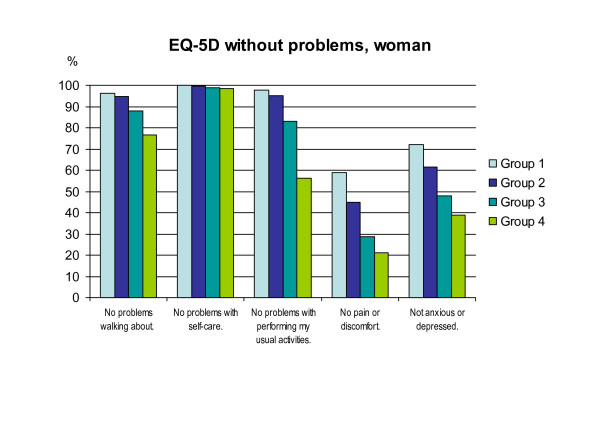
**Distribution in percent without any problems within the groups for women**.

**Figure 2:**
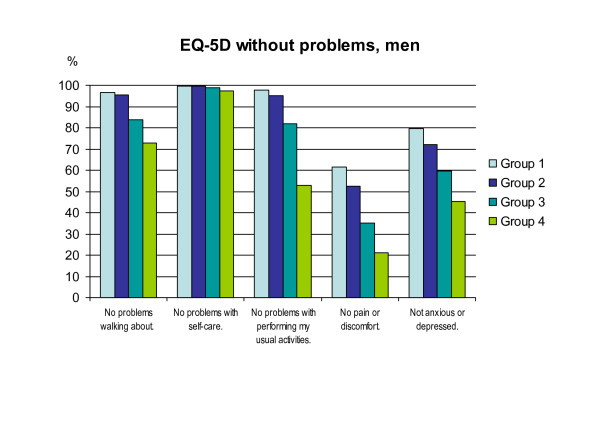
**Distribution in percent without any problems within the groups for men**.

Figure 1(women) and figure 2(men) shows the differences between the groups concerning the EQ-5D's five questions (one question for each of the dimensions of mobility, self-care, usual activities, pain/discomfort, anxiety/depression). There is a clear gradient with higher proportions of problems in group three and group four. The correlations between the groups and the EQ-5D questions, respectively, are all significant (p < 0.05, Spearman).

### Illnesses and complaints

Table 3 shows how long-term illnesses and complaints differ between the groups. The correlations between the groups and the illnesses are mostly significant (p < 0.01, Spearman). The ones that are not significant are Chronic Obstructive Pulmonary Disease for both sexes as well as Diabetes, Thyroid disorder, Food allergy and intolerance, Other skin disease, and Nickel allergy for men only.

**Table 3:** Distribution of percentage with reported long term illnesses and complaints during the last twelve months.

	Women				Men			
**Illnesses/Complaints**	Gr 1	Gr 2	Gr 3	Gr 4	Gr 1	Gr 2	Gr 3	Gr 4
Cardiovascular disease	0.8	1.4	4.0	4.8	2.1	2.3	9.4	9.2
Hypertension	9.6	10.6	16.8	16.9	9.7	10.2	22.4	27.7
Diabetes	1.9	1.8	4.3	4.7	3.0	2.2	5.5	6.8
Thyroid disorder	4.8	5.3	9.3	11.0	1.1	.8	1.9	3.7
Asthma	5.9	8.5	14.5	12.0	4.6	7.5	7.0	6.9
Allergic rhinoconjunctivitis	18.8	25.0	25.6	25.1	18.9	23.5	19.9	20.3
Food allergy and intolerance	7.4	8.8	9.8	10.0	4.2	5.0	5.4	6.7
Eczema	12.2	16.3	17.5	18.8	9.1	11.0	10.4	14.9
Other skin disease	4.7	6.4	6.1	6.9	6.0	6.9	6.6	9.2
Nickel allergy	11.5	13.6	19.3	15.2	1.7	2.3	2.5	0.0
Depression	6.3	10.4	27.1	37.4	4.5	7.4	20.6	28.3
Burn out syndrome	7.2	13.1	30.1	42.0	5.0	8.8	19.1	29.0
Cancer	0.7	0.6	2.7	8.1	0.3	0.4	4.8	4.8
Chronic obstructive pulmonary disease	0.4	0.4	1.1	1.4	0.3	0.4	0.4	0.6
Gastrointestinal disorder	7.1	9.5	16.9	14.1	5.5	9.3	21.2	12.6
Urinary incontinence	9.1	10.7	12.7	12.6	1.7	2.0	3.6	4.8
Rheumatoid arthritis	1.5	2.1	4.1	6.7	1.1	1.5	2.8	4.2
Neurological disorder	1.0	0.9	3.8	4.2	0.8	0.9	3.5	8.8
Sleep apnea syndrome	1.5	2.1	4.1	5.9	3.9	4.2	9.7	12.4
Tinnitus	7.1	8.7	15.7	12.7	13.7	15.9	18.0	22.3
Hearing disorder	7.2	10.1	10.4	14.2	16.4	18.6	25.1	25.3
Sight impairment despite glasses	1.8	2.4	3.3	3.2	2.9	3.6	6.9	5.6
Physical impairment	2.5	4.1	8.8	20.1	4.1	5.4	20.8	36.7
Psychological impairment	0.6	1.2	3.5	10.4	0.8	0.9	3.3	10.6

Age-standardised and weight-calibrated.

Table 4 shows how near-continuous complaints and symptoms for the last three months differ between the groups. The correlations between the groups and the complaints/symptoms are all significant (p < 0.01, Spearman).

**Table 4:** Distribution of reported near-continuous complaints and symptoms during the last three months.

	Women				Men			
**Complaints/Symptoms**	Gr 1	Gr 2	Gr 3	Gr 4	Gr 1	Gr 2	Gr 3	Gr 4
Pain in neck and shoulders	8.9	13.7	24.3	34.3	5.9	7.4	21.3	25.3
Pain in back or hips	5.0	8.2	16.7	23.4	4.4	6.6	12.6	23.9
Pain in arms, legs, knees or feet	6.3	8.4	19.7	23.8	4.4	5.7	16.7	23.1
Abdominal pain	1.1	1.7	2.5	2.5	0.5	0.9	4.2	2.4
Headache or migraine	0.9	2.3	3.7	6.0	0.7	0.7	2.9	5.0
Anxiety	1.3	2.1	7.0	11.3	1.0	1.6	5.3	7.3
Tiredness and weakness	2.1	4.2	12.0	20.7	1.2	2.4	7.7	15.5
Sleeping disorders	2.9	3.6	7.2	15.3	1.9	2.5	8.0	13.2
Depression	0.8	1.6	5.2	11.5	0.6	1.4	5.0	8.2
Dizziness	0.2	0.2	2.2	2.3	0.2	0.1	0.9	2.6
Irritated mucous membranes	0.8	1.3	2.1	3.3	0.9	1.1	1.5	1.7

Age-standardised and weight-calibrated.

### Diagnoses and sex

Two thirds of the subjects had both psychiatric and musculoskeletal symptoms. Among them there was a significant difference as men more often had reported musculoskeletal diagnoses. One third had only psychiatric or musculoskeletal symptoms and in those groups there were no significant differences in reported diagnoses between the sexes (Table 5).

**Table 5:** Crosstabulation of self-rated symptoms during past three months and diagnosis for respondents with more than 28 days of reported diagnosis for sickness absence during the past twelve months.

	Women		Men	
Self-rated symptoms	Psychiatric diagnoses	Musculoskeletal diagnoses	Psychiatric diagnoses	Musculoskeletal diagnoses
Psychiatric symptoms only	52	4	25	0
Musculoskeletal symptoms only	16	113	6	69
Psychiatric and musculoskeletal symptoms	189	195	36	95
Total	257	312	67	164

## Discussion

The aim of this study was to describe the association between degree of self-reported sickness absence and health. The Swedish social insurance system was at the time for the study based on the periods 0 days, 1–28 days, 29–89 days and 90 days or more of sickness absence. Due to these circumstances we divided the responders into these four groups. The hypothesis was that individuals with long-term sickness absence (more than 28 days) would report more symptoms and lower self-rated health. The area investigated covers 55 municipalities in central Sweden, with a total of about 1 million inhabitants in the age group. The response rate was deficient, only 65%. Another weakness of this population-based study is that it is founded on self-reported data only because we had no access to medical databases. To reflect the situation in the population as well as possible we decided to use calibrated and weighted data.

The results of the study describe self-reported sickness absence and health. Individuals with long-term sickness absence report more symptoms and lower self-rated health.

Men and woman sick-listed for 90 days or more generally reported a greater extent of illness and complaints compared to those who had been ill less than 90 days or who had not been absent at all. Unexpectedly, no sex differences for reported psychiatric and musculoskeletal diagnoses were found.

Another hypothesis was that women are more likely to self-rate psychiatric diagnoses compared to men, who are more likely to self-rate musculoskeletal diagnoses. Two thirds of the subjects had both psychiatric and musculoskeletal symptoms. Among them there was a significant difference as men more often had musculoskeletal diagnoses. One third had only psychiatric or musculoskeletal symptoms and in those groups there were no significant differences in diagnosis between the sexes. A possible explanation might be that physicians are more likely to diagnose women's symptoms as psychiatric and men's symptoms as musculoskeletal diagnosis.

Self-rated health and sickness absence has been evaluated in other studies [16].

Kivimäki et al found that a small amount of self-certified absence is protective. The Whitehall II study of British civil servants showed a strong association between indicators of ill health and sickness absence, particularly for longer spells of absence [17]. Self-rated health can be considered a relevant and important outcome measure for a patient-centred medical clinic. Overall self-rated health as measured by a single question proved to be significantly related to behavioural risk factors in a sample of primary care patients [18].

In our study there was a significant difference in self-rated health between the groups. With a greater extent of sickness absence a poorer self-rated health was reported.

The main differences observed between the groups in this report can be assumed to be reflected in health care utilisation and the extent of drug use. This can be investigated in further reports.

## Conclusion

Individuals with long-term sickness absence reported more symptoms and lower self-rated health than did those who had not been absent at all, and than those who had been ill 1–28 days. Men and women sick-listed 29 days or more generally reported more illness and complaints. Unexpectedly, no sex differences among psychiatric and musculoskeletal diagnoses were found.

## Competing interests

The authors declare that they have no competing interests.

## Authors' contributions

H-GE, A-SvC, LJ, RW and TW have made substantial contributions to conception and design. All authors have contributed with analysis and interpretation of data. H-GE have contributed by compiling data and producing tables and statistical analyses. All authors have been involved in drafting the manuscript or revising it critically. All authors read and approved the final manuscript.

## Pre-publication history

The pre-publication history for this paper can be accessed here:


